# Draft Genome Sequence of a Poly-γ-Glutamic Acid-Producing Isolate, Bacillus paralicheniformis Strain bcasdu2018/01

**DOI:** 10.1128/MRA.01013-21

**Published:** 2021-11-18

**Authors:** Anirban Adhikary, Tanu Bansal, Pooja Gupta, Deepti Jain, Purnima Anand, Rani Gupta, Jugsharan Singh Virdi, Ruchi Gulati Marwah

**Affiliations:** a Department of Microbiology, Bhaskaracharya College of Applied Sciences, University of Delhi, Dwarka, New Delhi, India; b Transcription Regulation Laboratory, Regional Centre for Biotechnology, Faridabad, Haryana, India; c Department of Microbiology, University of Delhi, South Campus, New Delhi, India; University of Rochester School of Medicine and Dentistry

## Abstract

Bacillus paralicheniformis bcasdu2018/01 was isolated from the indoor environment of a chemistry laboratory. As part of the extracellular matrix, this isolate produces copious amounts of poly-γ-glutamic acid (γ-PGA). Here, we report the 4.25-Mbp draft genome assembly of the organism with an average G+C content of 45.92%.

## ANNOUNCEMENT

The colony of Bacillus paralicheniformis bcasdu2018/01 was isolated on a nutrient agar (HiMedia Laboratories, India) plate exposed to the indoor environment of a chemistry laboratory of our institute in New Delhi, India. The Bacillus paralicheniformis group of bacteria are understudied from the perspective of biofilm formation and their capability to produce poly-γ-glutamic acid (γ-PGA) as a secreted biopolymer for industrial production. It is thus essential to identify a suitable genetic predisposition for its cost-effective production. Thus, we performed Illumina-based whole-genome sequencing of this bacterial isolate via Xcelris Labs (Ahmedabad, Gujarat, India).

Initially, the 16S rRNA gene was amplified using the primers 27F (5′-AGAGTTTGATCCTGGCTCAG-3′) and 1492R (5′-TACGGYTACCTTGTTACGACTT-3′) for sequencing using the Sanger method (GenBank accession number MK881510.2). A similarity search using the BLAST tool (1) against the NCBI rRNA database showed the closest similarity to Bacillus haynesii strain NRRL B-41327 (99.59%) and Bacillus licheniformis strain DSM-13 (99.46%). Genomic DNA was isolated using TRIzol reagent as per the manufacturer’s instructions. The quality of the isolated DNA was analyzed using agarose gel electrophoresis, and its concentration was determined using a Qubit 2.0 fluorometer. A TruSeq Nano DNA library prep kit was used to prepare the paired-end (PE) sequencing library. The library was sequenced using the Illumina HiSeq 2500 platform (2 × 150 bp chemistry), which generated a total of 141,059,626 reads with a coverage of 4,876×. The adapter sequences were removed and low-quality reads were filtered using Trimmomatic v0.36 with the following parameters: ILLUMINACLIP:adapter.fasta:2:30:10; LEADING:10; TRAILING: 10; SLIDINGWINDOW:0:20; MINLEN:40 (2). *De novo* assembly of the high-quality paired-end reads was accomplished using Velvet v1.2.10 (3), and the assemblies were optimized at kmer 121. GapCloser v1.12 software was used to remove gaps from the assembled scaffolds using PE read information (4). The final assembly consisted of 28 scaffolds, a genome size of 4,248,830 bp, an *N*_50_ scaffold value of 824,371 bp, an average scaffold length of 151,744 bp, a maximum scaffold length of 1,060,188 bp, a minimum scaffold length of 511 bp, and a G+C content of 45.92%. The assembled scaffolds were subjected to gene prediction using NCBI PGAP v5.2 (5). Similar entries to the predicted protein coding genes in the NCBI nonredundant (nr) database were identified using the BLASTP algorithm with an E value threshold of 1e-05. Similarity was also searched for against the UniProt, COG, and Pfam databases. Taxonomic identity was established by *in silico* DNA-DNA hybridization using formula 2 on the GGDC 2.1 server (6). Further confirmation was provided using ribosomal multilocus sequence typing (rMLST) (7). Secondary metabolites were predicted using antiSMASH v6.0 (8), and bacteriocins were predicted using BAGEL4 (9). Microsatellites were predicted using MISA-web (10), prophages using PHASTER (11, 12), and clustered interspaced short palindromic repeats (CRISPRs) using CRISPRfinder (13). The sequence identities of relevant genes were determined using the NCBI BLASTN tool. Default parameters were used for all software unless stated otherwise.

Gene prediction using PGAP identified a total of 4,278 genes comprising 4,108 protein coding sequences, 73 pseudogenes, 77 tRNA genes, 15 rRNA genes, and 5 noncoding RNA (ncRNA) genes. *In silico* DNA-DNA hybridization showed that the genome of this isolate had 90.6% similarity to the type strain Bacillus paralicheniformis KJ16, thus assigning it its species epithet. This finding was backed by rMLST analysis, which also identified it as Bacillus paralicheniformis. One putative CRISPR module, one intact and one incomplete prophage, and one simple sequence repeat (SSR) was found in the genome. Functional annotation against the UniProt database found the presence of the following genes involved in γ-PGA metabolism and secretion (BLASTN percentage identities to Bacillus licheniformis ATCC 14580 are indicated in parentheses): *pgsB* (97.72%), *pgsC* (98.22%), *pgsA* (96.58%), *pgsE* (96.43%), *pgdS* (94.38%), and *ggt* (94.8%). Apart from *ggt*, these genes are present in an operon. The genome also contains putative gene clusters for the production of secondary metabolites, namely, fengycin, lichenysin, bacteriocin, bacitracin, polyketides, bacilysin, petrobactin, streptin, bottromycin, lanthipeptides, and lasso peptides, and the siderophore bacillibactin.

### Data availability.

The whole-genome shotgun project has been deposited at GenBank under the accession number JAGTPZ000000000. The reads were deposited in the Sequence Read Archive (SRA) under the accession number SRX12486464. The BioProject accession number for the sequencing project is PRJNA724655.
